# Transthoracic echocardiography used in conjunction with passive leg raising for assessment of fluid responsiveness in severe sepsis or septic shock patients

**DOI:** 10.1186/cc13323

**Published:** 2014-03-17

**Authors:** K Jaikriengkrai, K Chittawatanarat, A Limsukon

**Affiliations:** 1 Chiang Mai University, Chiang Mai, Thailand

## Introduction

During passive leg raising (PLR), we need a real-time device to demonstrate the hemodynamic change [1-3]. This study investigates the ability of transthoracic echocardiography (TTE) to predict fluid responsiveness (FR) in terms of detecting change of stroke volume (ΔSV) after PLR compared with the transpulmonary thermodilution technique (TPTD) ΔSV after volume expansion (VE).

## Methods

A prospective study was carried out in a medical ICU. Eligible patients were age ≥18 years without necessarily full adaptation to the ventilator with hemodynamic instability who were considered for VE. SV assessment using the subaortic velocity-time measurement was obtained by TTE simultaneously with other hemodynamic parameters derived from TPTD at baseline, within 2 minutes of PLR and following VE (250 ml fluid in 10 minutes). A fluid responder was defined by ΔSV ≥15% after VE by TPTD.

## Results

**Figure 1:**
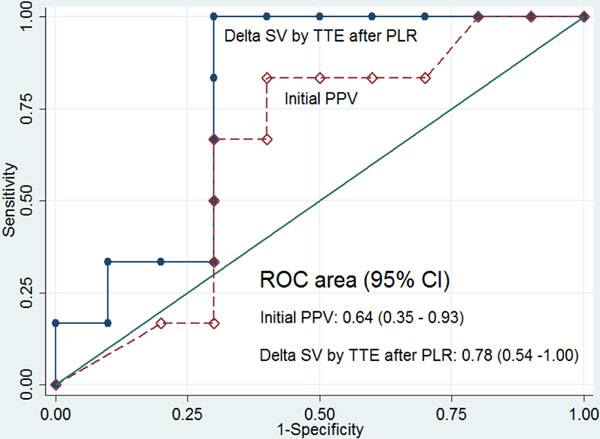
**ΔSV-TTE after PLR≥13.6% may predict fluid responsiveness with sensitivity of 83.33%, specificity of 70% and AUC of 0.78 (95% Cl: 0.54 to 1.00). Initial PPV ≥11% predicted fluid responsiveness with sensitivity of 83.33%, lower specificity of 60% and AUC of 0.64 (95% Cl: 0.35 to 0.93)**.

**Figure 2:**
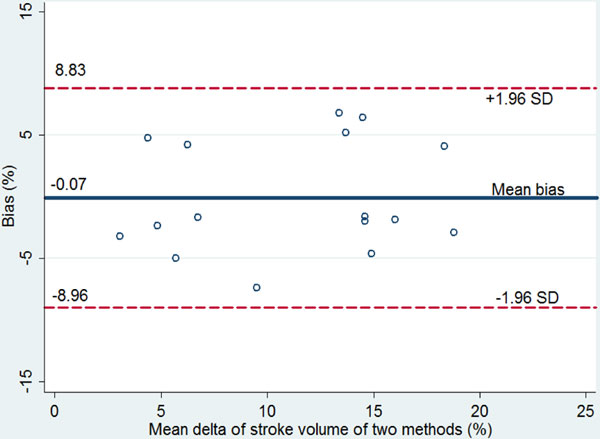
**Bland-Altman plot, comparing two methods, showing 95% limits of agreement from -8.96 to +8.83%ΔSV and the mean difference (bias) of measurement is -0.07%ΔSV. Dashed lines, upper and lower limits of agreement (95% Cl for repeated measurements)**.

Preliminary reports on 16 patients with satisfactory cardiac windows were analyzed. ΔSV-TTE after PLR ≥13.6% predicts FR with sensitivity of 83.33%, specificity of 70% and AUC of 0.78 (95% CI: 0.54 to 1.00). Initial PPV ≥11% predicted FR with sensitivity of 83.33% with lower specificity of 60% and AUC of 0.64 (95% CI: 0.35 to 0.93) (Figure 1). The Bland-Altman plot showed 95% limits of agreement from -8.96 to +8.83% and mean difference (bias) of -0.07% (Figure 2).

## Conclusion

We may use %ΔSV measured by TTE after PLR to predict FR, which is noninvasive and less time-consuming than other invasive techniques.
